# Microsolvation Controls Ultrafast Relaxation Pathways in Hydrated Pyridine

**DOI:** 10.1021/acs.jpca.6c05980

**Published:** 2026-10-07

**Authors:** Deepthy Maria Mootheril, Anna D. Skitnevskaya, Xueguang Ren, Alexander I. Kuleff, Alexander B. Trofimov, Lorenz S. Cederbaum, Thomas Pfeifer, Alexander Dorn

**Affiliations:** † 28297 Max-Planck-Institut Für Kernphysik, Saupfercheckweg 1, 69117 Heidelberg, Germany; ‡ Federal Research Center, 54743 A.E. Favorsky Irkutsk Institute of Chemistry, Siberian Branch of the Russian Academy of Sciences, 664033 Irkutsk, Russia; § MOE Key Laboratory for Nonequilibrium Synthesis and Modulation of Condensed Matter, School of Physics, 12480 Xi’an Jiaotong University, Xi’an 710049, China; ∥ Theoretische Chemie, Physikalisch-Chemisches Institut, 9144 Universität Heidelberg, 69120 Heidelberg, Germany

## Abstract

Hydration critically reshapes ultrafast electronic relaxation in biorelevant molecules, yet its mechanistic role remains poorly understood. Here, we investigate intermolecular Coulombic decay (ICD) following medium-energy (*E*_0_ = 103 eV) electron impact ionization of hydrated pyridine, a prototypical nucleobase analogue. Combining multiparticle momentum coincidence spectroscopy with high-level electronic structure calculations, we identify water-initiated ICD and find evidence consistent with an additional pyridine-initiated ICD pathway, in which inner-valence ionization leads to ionization of the neighboring molecule. Water vacancies decay exclusively via the ICD, establishing a highly efficient direct channel. In contrast, pyridine vacancies compete with Auger decay, reducing the efficiency of the pyridine-to-water ICD pathway. As in pyridine dimers, heterocycle-initiated ICD is dominated by N 2s vacancy states. Our results reveal a dual role of water: it opens a highly efficient direct ICD pathway via O 2s vacancy states and acts as an energy-accepting partner following biomolecular inner-valence ionization. These results reveal how microsolvation governs ultrafast relaxation and shapes radiation-induced damage pathways in DNA-related systems.

## Graphical abstract

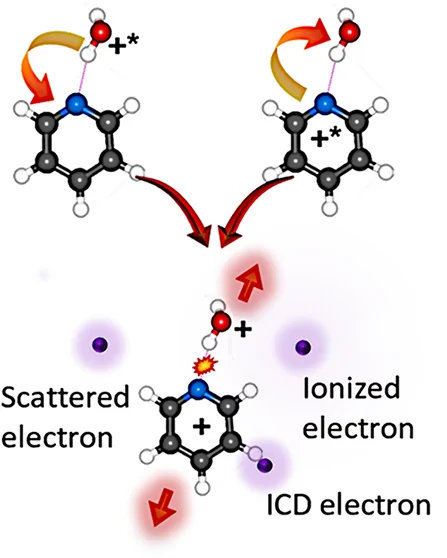

## 1 Introduction

Hydrogen-bonded systems are ubiquitous in nature as biological matter is largely embedded in water. Studies of clusters and complexes of biomolecules involving hydrogen bonds provide significant insights into the mechanisms of radiation-induced damage, particularly in DNA.^1^ Such investigations are relevant to fields ranging from fundamental physics to cancer therapies. In weakly bound molecular environments, the creation of an inner-valence or a core vacancy often initiates local or nonlocal relaxation processes that redistribute the excess energy to neighboring molecules.^2^ Such environments are prevalent in biological systems, where water molecules form extensive hydrogen-bonded networks surrounding biomolecules.^3^ Following inner-valence or core ionization of water, both local and nonlocal relaxation mechanisms can transfer energy and charge to nearby biomolecules, potentially leading to their ionization and fragmentation. A prominent example of such a nonlocal relaxation pathway is the intermolecular Coulombic decay (ICD),^4^ where the excess energy released from the relaxation of a molecular inner-valence vacancy is transferred to a neighboring molecule ionizing it, producing in that way two positive charges on two nearby centers.^5^

Hydrogen bonding, being directional and stronger than van der Waals forces, profoundly influences the structural and electronic properties of molecular aggregates and therefore may also affect the ICD process.^6^ Understanding ICD in hydrogen-bonded systems is essential for elucidating energy dissipation mechanisms in biologically and chemically relevant environments such as aqueous solutions and hydrated biomolecules. ICD has been established as a major source of low-energy electrons (LEEs) and reactive fragments in aqueous environments,^7^ with implications in radiation-induced biomolecular damage.^1^ Recent studies have further shown that in small water clusters, proton transfer can compete with and, in certain cases, even suppress ICD,^8^ leading to alternative fragmentation pathways.

Beyond pure water, ICD has also been experimentally studied in hydrogen-bonded biomolecular complexes. For example, in the water-THF (tetrahydrofuran) complex, ICD from the oxygen inner-valence states of water to THF was found to dominate, while ICD originating from THF was suppressed by competing Auger decay channels, a process involving the ejection of a second electron from the same THF molecule.^9^ The THF–water complex is particularly relevant to DNA-damage studies, as THF serves as a molecular analogue of the deoxyribose unit in DNA. Similarly, X-ray-induced core photoionization of water-pyrimidine complexes revealed that the surrounding environment enhances the ICD rate from pyrimidine to water, thereby protecting the biomolecule from fragmentation by dissipating the energy into the environment. On the contrary, ICD from water to pyrimidine was shown to transfer energy from the environment to the heterocycle ionizing it; however, presumably nondissociative cationic states were formed.^2,10^ Heavy-ion collisions on pyrimidine-water complexes have further demonstrated that ICD can also be triggered by particle impact, providing a robust energy dissipation channel in hydrated nucleobase analogues.^11^

Here, we extend this line of investigation to hydrated pyridine, another biologically relevant molecular complex containing nitrogen. Pyridine is a heteroaromatic compound with a six-membered ring structure comprising one nitrogen atom, five carbon atoms, and five hydrogen atoms. The aromatic system in pyridine is formed by six π-electrons: one from each carbon and one from the sp^2^-hybridized nitrogen. ICD has been previously observed in pyridine dimers, initiated from the C 2 s^–1^ and N 2 s^–1^ inner-valence vacancies above ∼25 eV, as well as in other dimers of nitrogen-containing heterocycles. The Coulomb explosion imaging technique was exploited to study how the number and position of nitrogen atoms within six-membered aromatic ring molecules influence the dimer structures and their bonding type.^12^ These heterocycles involving sp^2^-hybridized nitrogen can form hydrogen bonds with water molecules. Such interactions are highly relevant to radiation damage studies, as the aromatic rings of DNA bases contain nitrogen atoms that can form hydrogen bonds with surrounding water molecules.

Here, we investigate the ICD process in the pyridine-D_2_O complex, which serves as a prototypical hydrogen-bonded complex. Pyridine forms a well-defined hydrogen bond with water via its nitrogen lone pair that acts as an electron density donor.^6^ Although much simpler than hydrated nucleobases, this complex retains key features of nitrogen-containing aromatic molecules interacting with water and allows systematic investigation of the role of hydrogen bonding in facilitating nonlocal relaxation. Deuterated water was used instead of H_2_O to improve mass separation in the time-of-flight spectrum of the resulting fragments. While deuteration does not alter the electronic structure and, therefore, the electronic decay processes investigated here, it can affect nuclear dynamics-driven processes through isotope effects. In particular, processes such as proton/deuteron transfer may occur on different time scales owing to the larger mass of deuterium. Since the present work focuses on the electronic decay pathways and their signatures in the electron and ion spectra, such nuclear dynamic effects are not explicitly considered.

## II Methods

### II.I Experimental Methods

The experiment was conducted using a reaction microscope (ReMi),^13^ specifically designed for electron impact ionization studies. A detailed description of the apparatus is available elsewhere.^14^ Pyridine-D_2_O complexes were prepared using a supersonic gas jet. Helium gas at 1 bar, used as the carrier gas, was guided through the first reservoir containing D_2_O, which is heated to approximately 52 °C to achieve a vapor pressure of around 140 mbar.^15^ This mixture of helium gas and D_2_O vapor was then passed through a second reservoir, heated to 62 °C, containing pyridine at a vapor pressure near 170 mbar.^16^ The resulting gas mixture was expanded through a nozzle with a diameter of 30 μm, where rapid adiabatic cooling and three-body collisions with helium facilitate the formation of pyridine-D_2_O complexes. As a result, the jet contained mixed clusters of varying sizes. The pyridine-D_2_O complex-containing gas jet was ionized by an electron beam with 103 eV energy and a pulse duration of 0.5 ns. The electron beam was generated by a photoemission electron gun and focused to a diameter of approximately 1 mm at the interaction region. The beam was aligned along the spectrometer axis, and the unscattered projectiles were dumped into a central bore in the detector.

The resulting charged fragments (electrons and ions) were extracted from the interaction region using homogeneous electric and magnetic fields and projected onto time- and position-sensitive detectors placed on opposite sides of the spectrometer. Electrons were guided by a 3 V/cm electric field in combination with a 6.9 G magnetic field. For ion extraction, the magnetic field was kept constant, while the electric field was increased to 22.6 V/cm about 1 μs after electron detection. From the measured time-of-flight (TOF) values and detector hit positions, the momentum vectors of the particles were reconstructed. Helium, used as the carrier gas, also served to calibrate the momentum analysis. The error bars on the data points shown below indicate statistical uncertainties. The energy resolution of the detected electrons depends on their energy and momentum, as well as on the applied electric and magnetic fields. For the fast scattered projectile electrons, the energy resolution is approximately 6 eV, whereas for the slow ejected and ICD electrons, it is less than 1 eV.

### II.II Computational Methods

The molecular structures analyzed in this work were optimized at the MP2 level of theory with the aug-cc-pVDZ basis set as implemented in the Gaussian16 package.^17^ In our calculations, H_2_O has been used instead of D_2_O, as the two isotopologues do not differ in their stationary geometries or electronic structure. Since no dynamics simulations have been performed, this substitution has no effect on the reported results. The obtained mutual orientation of the molecules in the pyridine-H_2_O dimer is consistent with previously reported studies.^18,19^ Ionization spectra were then computed using the third-order Algebraic Diagrammatic Construction scheme ADC(3) to approximate the one-particle propagator for single ionization potentials (IPs),^20^ and second-order ADC(2) to approximate the two-particle Green’s function for double-ionization potentials (DIPs),^21,22^ both employing the aug-cc-pVDZ basis set. The eigenvalue problem associated with the ADC­(*n*) Hamiltonian matrices was solved using the Block-Lanczos algorithm.^23–25^ Finally, DIP states were assigned as either localized, with two holes residing on one of the molecules, or delocalized, with holes shared between pyridine and H_2_O, using the two-hole population analysis method integrated with the DIP-ADC(2) framework.^26^

To improve comparison with experiment and account for the intrinsic limitations of the calculations, empirical energy shifts were applied to the computed ionic states. The calculated ADC(2)/aug-cc-pVDZ DIPs were blue-shifted by 1 eV. This is an averaged value evaluated by comparison of the theoretical thresholds computed in this work with established experimental values: 24.8 eV vs 25.5 eV^27^ for pyridine double ionization and 38.7 eV vs 40.0 eV^28^ for water double ionization. The ADC(3)/aug-cc-pVDZ single ionization spectrum of pyridine requires red shifts of 1.2 and 1.5 eV for the C 2 s^–1^ and N 2 s^–1^ states, respectively, and a shift of 1.1 eV for the O 2 s^–1^ (2a_1_^–1^) state of water. These values were determined by aligning the theoretical spectra of pyridine and water molecules with corresponding experimental spectra^29,30^ (see more details and figures in SM, Section 2). Although the applied shifts exceed the typical mean error of the ADC(3) method^31^ known for the valence region, they are justified by the strong contribution of 2h-1p (two-hole-one-particle) states in the inner-valence region addressed here.^32^ Within the IP-ADC(3) framework, such states are only considered at first order of perturbation theory, which leads to an increase in computational error.

The secondary-electron energy distributions were obtained from the energies of electrons emitted in all energetically allowed transitions to delocalized dicationic states, assuming that the electron kinetic energy equals the energy difference between the initial (IP) and final (DIP) states. The overall procedure follows refs 33,34 . This data was used to build a three-dimensional (3D) map as described in ref^35^ using fwhm = 0.3 eV. The map is shown in Figure 4(c) and presents the distribution of the ICD electron energies as a function of the initial states. The secondary-electron energy distribution was also plotted as two-dimensional (2D) spectra to allow comparison with experimental data in Figure 3b. Only IP states with energies above 26.5 eV, which corresponds to the experimentally evaluated ICD threshold, were considered. Secondary electrons with energies below 0.5 eV were excluded from the resulting distribution to allow a proper comparison with experiment, where electrons of such energies are not detected. The contributions to the secondary ICD electron spectrum from different IP bands were computed separately, and the total spectra were then plotted using different ratios to enable analysis of the influence of pyridine and water decay on the shape of the spectrum (Figure 3(b)); see the Discussionsection for details.

## III Results

We study the Coulomb explosion (CE) of pyridine-D_2_O dimers following electron impact ionization. The process can be summarized as

1 $$\begin{array}{l} e^{-}\left(E_{0}=103\;eV\right)+C_{5}H_{5}N\cdot \cdot \cdot D_{2}O\to {\left(C_{5}H_{5}N\cdot \cdot \cdot D_{2}O\right)}^{+*}+2e^{-}\\ \to C_{5}H_{5}N^{+}+D_{2}O^{+}+2e^{-}+e_{\mathrm{ICD}}^{-} \end{array}$$

Among the five final-state particles, both ionic fragments undergoing CE are detected in coincidence with one of the outgoing electrons. Such CE events are identified in the ion–ion time-coincidence map shown in Figure 1(a), within the black dashed rectangle. The surrounding background originates from several processes: (1) false coincidences of C_5_H_5_N^+^ with D_2_O^+^ or OD^+^, together with unrelated ions, appear as a horizontal line at 13,850 ns and as vertical lines at 7325 and 7700 ns, respectively. (2) A broad and intense distribution on the right side of the CE channel of C_5_H_5_N^+^ + D_2_O^+^ arises from D_3_O^+^ + C_5_H_5_N^+^, which originates from larger clusters containing more than one D_2_O molecule.^11,36^ In this case, D_3_O^+^ is produced by deuteron transfer from neighboring D_2_O^+^. The coincidence distribution appears to be broadened because neutral fragments carry away part of the momentum. (3) Finally, a faint but sharp line to the right of the C_5_H_5_N^+^ + D_2_O^+^ CE channel suggests proton transfer from pyridine to D_2_O prior to CE, producing D_2_OH^+^ and C_5_H_4_N^+^ ions.

**Figure 1:**
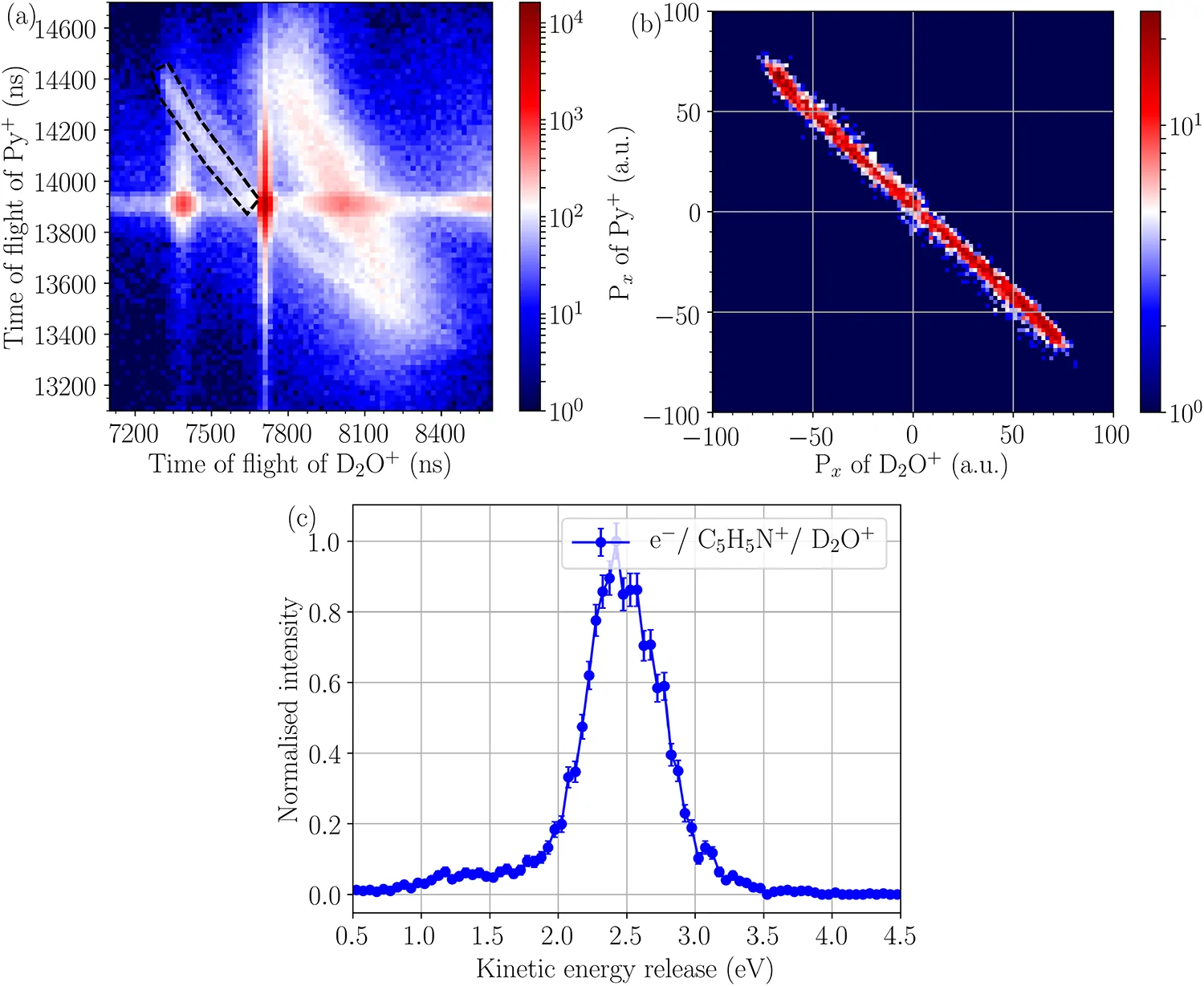
Time-of-flight and momentum coincidence map of the Coulomb explosion fragments and their corresponding KER spectrum. (a) Time-of-flight coincidence map. (b) Momentum coincidence map of ions in the *x* direction from the CE events of the pyridine-D_2_O complex after background separation. (c) Kinetic energy release (KER) spectrum corresponding to the C_5_H_5_N^+^ and D_2_O^+^ CE events.

In the following, we focus on the CE channel producing C_5_H_5_N^+^ and D_2_O^+^. The corresponding coincidence line in Figure 1(a) extends downward and overlaps with the broad distribution from D_3_O^+^ and C_5_H_5_N^+^ (process 2). To isolate the CE channel, we consider only events where C_5_H_5_N^+^ is emitted in the +*z* direction, as indicated within the black dashed rectangle of Figure 1(a). A slight tilt of the coincidence line in Figure 1(a) results from inhomogeneity in the spectrometer electric field during the ramp-up phase of ion extraction. This effect is corrected during momentum reconstruction, with details of the algorithm provided in the SM, Section 1. CE events are distinguished from background contributions by selecting ion pairs that satisfy momentum conservation. The details of the filtering procedure are described in ref 37

### III.I Momentum Coincidence Map of the CE Fragments and the KER Spectrum

The momentum coincidence map in the *x* direction of C_5_H_5_N^+^ and D_2_O^+^ ions after the background removal is shown in Figure 1(b). The corresponding kinetic energy release (KER) distribution of the CE channel is shown in Figure 1(c). The KER spectrum shows a broad distribution with a low-energy shoulder. Since the C_5_H_5_N-D_2_O dimer has one preferred conformational geometry,^19^ the main peak should correspond to the equilibrium intermolecular separation. The low-energy shoulder indicates a fraction of CE occurring at a larger intermolecular distance. This could arise from nuclear dynamics occurring after inner-valence ionization and on a time scale comparable with the ICD, or from the redistribution of the energy within the fragments’ internal degrees of freedom during Coulomb explosion (e.g., rotation^38^).

From the momentum and energy information on the electron detected in coincidence with the ions, the experimental ICD onset energy and the ICD electron energy spectrum can be obtained. The final-state electron energy spectrum ranges from 0 eV up to (*E*_0_ – *E*_bind_), where *E*_0_ is the projectile energy and *E*_bind_ is the binding energy of the ionized orbital. With high propencity, the low-energy part of the electron spectrum can be assigned to the ejected electron and the ICD electron, while the high-energy part is formed from scattered projectiles.

### III.II Possible ICD Pathways

To identify the molecular center and the inner-valence cationic state from which ICD is initiated, we examine the projectile energy-loss spectrum with the energy loss defined as *E*_0_ – *E*_1_ (*E*_1_ is the scattered projectile energy). The onset of this spectrum measured in coincidence with the ion pair corresponds to the lowest in energy inner-valence cationic state for which the ICD process is open. The blue open circles in Figure 2 show the projectile energy-loss spectrum for the CE channel under investigation. A broad onset is observed around ∼26.5 eV. The width of this onset arises from the energy resolution of the scattered projectile (∼6 eV), as well as from the broadening due to Franck–Condon overlap between different vibrational states, as discussed in detail in ref 37. The instrumental resolution can be seen from the onset of the energy-loss spectrum for helium ionization, which is also shown in Figure 2(a).

**Figure 2:**
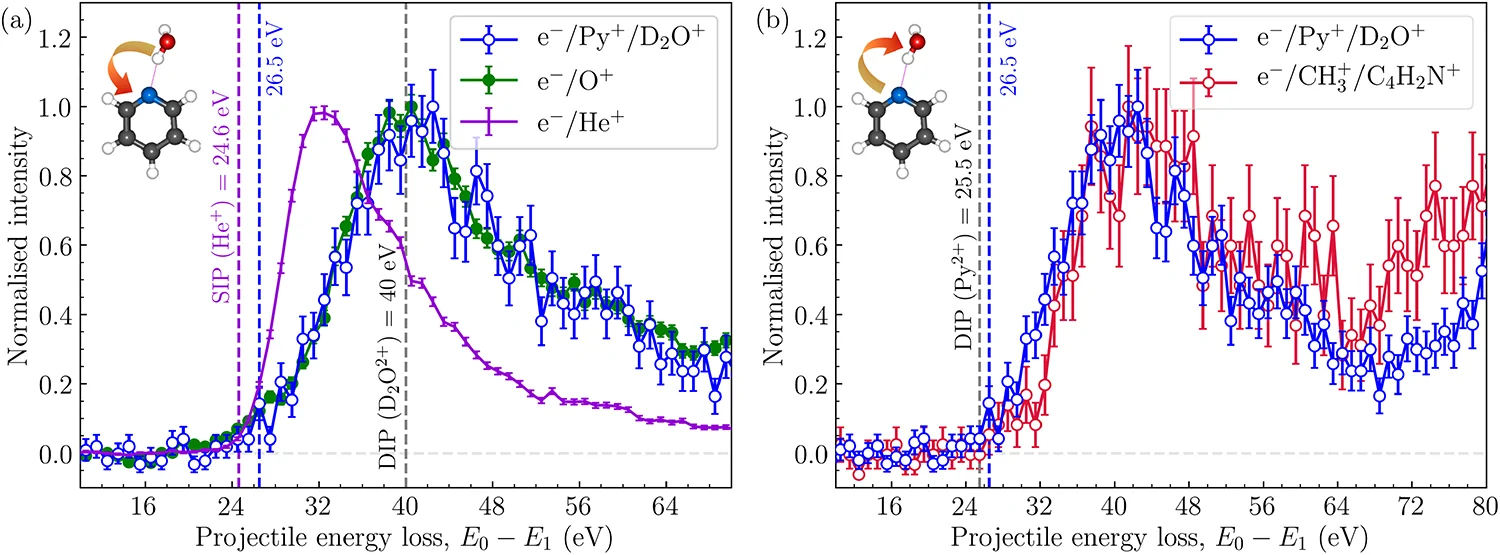
Projectile energy-loss spectra for various monomer ionization channels in comparison with the CE channel. (a) Energy-loss spectra for the CE channel of the pyridine-D_2_O complex (blue open circles), D_2_O inner-valence ionization leading to O^+^ (green solid circles), and helium single ionization (violet line). The violet dashed vertical line at 24.6 eV shows the helium single ionization potential. The gray dashed vertical line indicates the double-ionization threshold of the isolated D_2_O molecule. (b) Energy-loss spectra for the CE channel of the pyridine-D_2_O complex (blue open circles, same as in (a) but up to higher energies) and for pyridine monomer inner-valence ionization leading to Auger decay and subsequent fragmentation into CH_3_^+^ and C_4_H_2_N^+^ ions (red open circles). The gray dashed vertical line indicates the double-ionization threshold of the isolated pyridine molecule. The blue dashed vertical line at 26.5 eV shows the binding energy of the CE channel, the D_2_O monomer ionization channels, and the pyridine fragmentation channel.

In a C_5_H_5_N-D_2_O dimer, two ICD pathways are possible. The process can be initiated either by an inner-valence ionization of D_2_O, leading to energy transfer from D_2_O to pyridine, or by an inner-valence ionization of pyridine, leading to energy transfer from pyridine to D_2_O. The possibility and efficiency of the first pathway are supported by the fact that the experimentally observed ICD onset lies at the lower-energy edge of the O 2 s^–1^ band. The O 2 s^–1^ has a main line at approximately 32.2 eV and a lower-energy shoulder consisting of shake-down satellite states around 27.6 eV.^30^ At the same time, the double-ionization threshold of D_2_O (40 eV^28,33^ ) is significantly higher than the O 2 s^–1^ band position; thus, the usual ICD competitor, Auger decay, is energetically closed. For the second pathway, by contrast, the observed ICD onset lies above the DI threshold of pyridine, which is experimentally evaluated to be 25.5 eV.^27^ Ionization of the heterocycle inner-valence orbitals beyond the DI threshold can, therefore, lead to Auger decay and fragmentation in the case of the pyridine monomer, while in the C_5_H_5_N-D_2_O dimer, both ICD and Auger channels are accessible and may compete.^6^ Therefore, ICD pathways are possible following inner-valence ionization of both D_2_O and pyridine, but it should occur more efficiently in the former.

To demonstrate that the O 2s vacancy decays via ICD in the presence of a neighbor, we compare the projectile energy-loss spectrum of the D_2_O monomer ionization channel originating from the O 2s orbital with that of the CE channel of the pyridine-D_2_O complex. Previous photoionization measurements on isolated D_2_O monomers show that ionization of the O 2s electron leads to dissociation, producing O^+^, OD^+^, and D^+^ with branching ratios of 24%, 16%, and 60%, respectively.^39^ For the monomer case, we select events from the dissociation channel producing O^+^ ions, as those are observed exclusively after O 2s ionization. The green solid circles in Figure 2(a) show the corresponding energy-loss spectrum. The onset of this spectrum closely matches that of the CE channel, confirming that ionization of the O 2 s^–1^ inner-valence orbital in D_2_O can transfer energy to pyridine via ICD.

As described above, the possibility of ICD initiated by a pyridine inner-valence vacancy around 26 eV cannot be excluded. Since Auger decay channels are also energetically accessible in this energy range, an isolated pyridine monomer is likely to become doubly charged and subsequently dissociate. As an example, we consider the dissociative double-ionization channel producing CH_3_^+^ and C_4_H_2_N^+^ ions to illustrate one possible decay pathway following inner-valence ionization in this energy region.^40^ Several other fragmentation channels may arise from the decay of inner-valence-ionized states; the present channel is selected because it exhibits minimal overlap with other channels in the time-coincidence map, thereby facilitating a more reliable analysis. Figure 2(b) compares the energy-loss spectra of the above-mentioned monomer fragmentation channel and the ICD channel. The similar onset energies show that pyridine inner-valence states associated with dissociative double ionization are in the energy range where ICD is energetically open and can therefore serve as potential ICD initiators. The contribution of the pyridine inner-valence states to the observed ICD signal is further supported by the ICD electron spectrum and its comparison with the calculated electron energy distributions discussed below. In the pyridine-D_2_O dimer, the corresponding intermediate singly ionized excited state, Py^•+^-D_2_O, can in principle decay either via Auger decay or ICD. In a possible Auger pathway, the pyridine molecule becomes doubly ionized and may fragment, whereas the D_2_O molecule remains neutral. Alternatively, ICD from this state would transfer the excess energy to the neighboring D_2_O molecule, leading to its ionization and resulting in the Py^+^+D_2_O^+^ final state.

### III.III Extraction of ICD Electron Energy Spectrum

In the following discussion, we extract the ICD electron energy spectrum by comparing the low-energy electron distributions of the ICD channel to those of the D_2_O monomer dissociation channel. For the monomer ionization channel producing O^+^, the electron energy spectrum (red open circles in Figure 3(a)) consists of low-energy ejected electrons and fast scattered projectile electrons at energies above approximately 21 eV. In contrast, the spectrum associated with the ICD channel (black open circles in Figure 3(a)) contains contributions from scattered and ejected electrons originating from O 2s and N 2s ionization, in addition to low-energy ICD electrons emitted from both water and pyridine.

**Figure 3:**
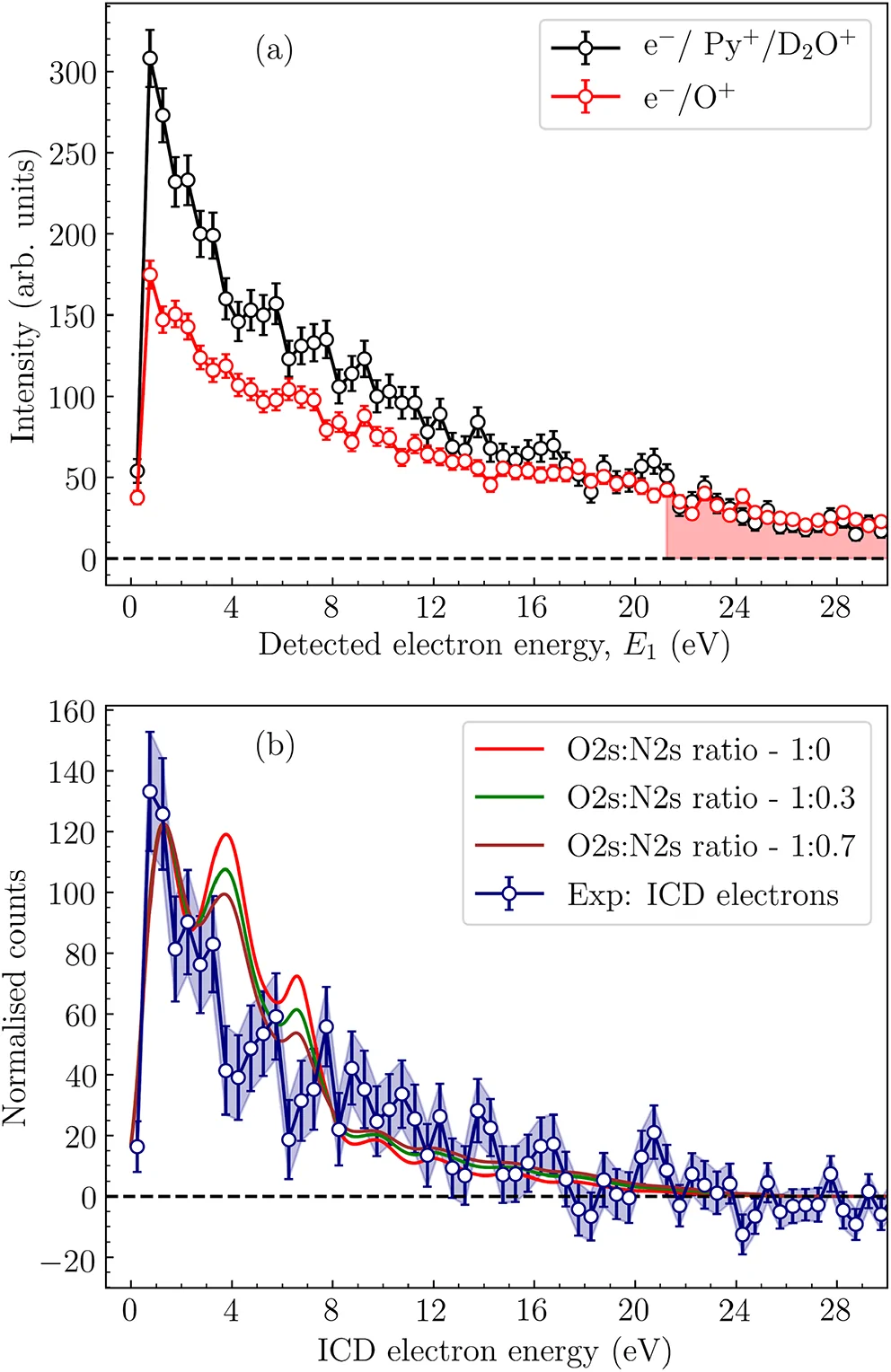
Experimental ICD electron energy spectrum. (a) Ejected electron energy spectra for the CE channel and the D_2_O monomer inner-valence ionization channel. Both spectra are normalized above 21 eV, as the region below 21 eV is expected to show an enhancement due to ICD electrons. (b) The ICD electron spectrum obtained as the difference between the two experimental curves of panel (a) is shown in blue. The red, green, and brown curves show the theoretically computed ICD electron energy distributions with different relative contributions of O 2s and N 2s vacancy decay; see the Methodssection for computational details.

The enhancement of low-energy electrons in the ICD spectrum relative to that in the monomer dissociation channel is therefore attributed to ICD electrons. To extract the ICD electron distribution, we must remove the contributions from the scattered projectile and the primary ejected electron. The O^+^ dissociation channel can be used as a reference spectrum for this purpose, provided that the energy distributions of the scattered projectile and the primary ejected electron are similar for inner-valence ionization from both pyridine and D_2_O. Since the scattered projectile and the ejected electron share the excess energy available after ionization, their energy distributions are related.^41^ The close similarity of the scattered projectile energy distributions for the pyridine dissociative double ionization and the O 2s ionization channel (Figure 2) therefore suggests that the corresponding primary ejected electron distributions are also comparable.

Consequently, the ICD electron energy distribution is obtained by comparing the electron spectrum of the O^+^ dissociation channel with that of the ICD channel. Both spectra are normalized to their integrated intensity above 21 eV, where only scattered projectiles and primary ejected electrons are expected, and ICD electrons do not contribute. Subtracting the normalized spectra yields the ICD electron energy distribution shown in blue open circles in Figure 3(b). The detection efficiency decreases sharply for electrons with energies below 0.5 eV because of the central bore in the detector. Possible differences in angular distributions, detection efficiencies, or residual contributions from unresolved fragmentation channels may contribute to systematic uncertainty and the shape of the extracted ICD spectrum.

### III.IV Theoretical Spectra of Pyridine-D_2_O Complex and Comparison with the Measured ICD Electron Energy Spectrum

Figure 4 shows the theoretical spectra of the pyridine-D_2_O complex. Figure 4(a) shows the double-ionization spectrum with localized, one-site (orange), and delocalized, two-site (blue) doubly charged states depicted separately. The localized states can be produced via the Auger decay of the singly charged states located on pyridine. An alternative pathway for the formation of pyridine dications is through electron transfer mediated decay (ETMD).^42^ This process can occur following single inner-valence ionization of water, where the initial vacancy is filled by an electron from pyridine, accompanied by the emission of a second electron from pyridine. Although the ETMD channel is energetically open for an inner-valence vacancy in water within the C_5_H_5_N-D_2_O complex, it is not expected to contribute significantly. This is because ETMD must compete with the ICD of the O 2s vacancy, which is generally much faster, as the ETMD rate is limited by the relatively slow intermolecular electron transfer step.^5^

The delocalized states can be produced via ICD from any inner-valence state above the ICD threshold. According to our theoretical calculations, this threshold is approximately 24 eV. In this energy range, the theoretical single ionization spectrum (Figure 4(b)) contains three possible ICD initiators: the C 2s and N 2s vacancies of pyridine and the O 2s vacancy of D_2_O. However, the experimentally observed ICD threshold, determined from the onset of the projectile energy-loss spectrum (Figure 2), lies at approximately 26.5 eV and is therefore higher than the theoretical prediction. This suggests that C 2s vacancies, which could undergo ICD above 24 eV, do not contribute significantly to this process. The experimentally observed threshold is consistent with ICD originating from the N 2 s^–1^-dominated band above 26 eV. While these states can also decay via the usually faster Auger process, producing C_5_H_5_N^2+^, they may also undergo ICD, as previously reported for the pyridine dimer.^12^

The O 2s vacancy of D_2_O is located above 28 eV, with the main band at 30–32 eV, and does not have competing Auger channels. Thus, these states decay predominantly via ICD and provide the dominant contribution to the formation of CE products C_5_H_5_N^+^ and D_2_O^+^. These comparative analyses of the calculations and the experimental results suggest that ICD from D_2_O is the dominant pathway in the pyridine-water dimer.

Figure 4(c) presents the theoretical secondary ICD electron energy distribution as a function of the ionization energy of the system. The 3D spectrum was generated in a manner similar to the electron–electron coincidence spectrum reported in ref^35^ from the IP and DIP spectra by considering all energetically allowed transitions from each IP state to the accessible delocalized DIP states. The transition probabilities were approximated by the product of the intensities of the corresponding initial and final states without any additional scaling. ICD efficiencies for all inner-valence bands were assumed to be equal. The same data is used to build theoretical ICD electron energy spectra with different relative contributions of the O 2s and N 2s vacancies, which are shown as the red, green, and brown curves in Figure 3(b). From Figures 4(c) and[Fig fig3](b), one can see that the O 2s vacancy gives rise to the broad secondary-electron energy distribution, spanning energies up to approximately 8 eV. At low energy, up to 5 eV, it does not reproduce the experimental spectrum, which shows a dominant maximum at around 1 eV. Additional intensity at this energy range can only be reproduced by adding the contributions from the decay of the 26–30 eV band, which is dominated by the N 2 s^–1^ contributions (green and brown curves in Figure 3(b)). Thus, pyridine accounts for the pronounced low-energy enhancement observed in the experimental ICD electron spectrum shown in Figure 3(b). The resulting ICD electron spectrum shows reasonable agreement with the experimental data.

**Figure 4:**
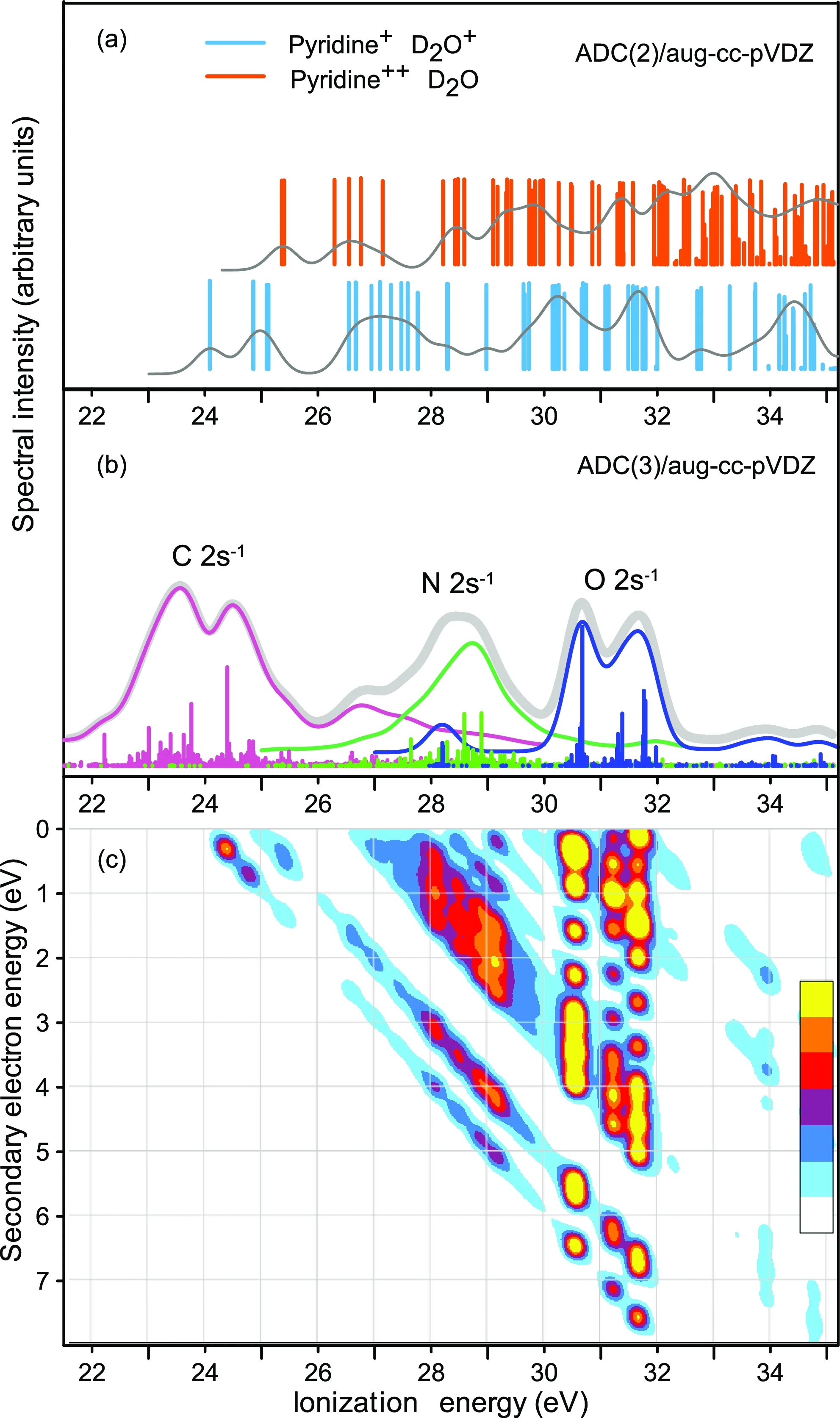
Theoretical spectra of pyridine-D_2_O complex. (a) Double-ionization spectra (DIP). Delocalized states with one vacancy on pyridine and one on water are shown in blue, while localized states with both vacancies on pyridine are shown in orange. The gray curves represent the convolution of the stick spectra. (b) Inner-valence region of the single ionization spectra (IP). Vertical bars and colored envelopes represent the contributions of the individual C 2 s^–1^ (purple), N 2 s^–1^ (green), and O 2 s^–1^ (dark blue) cationic states. The total envelope is shown in gray. All IP spectra are convoluted with a Gaussian of fwhm = 0.6 eV. (c) Theoretical ICD electron energy distribution as a function of the system ionization energy. The intensity is color-coded from white (zero) to yellow (maximum).

Possible competing channels of N 2s vacancy relaxation include Auger decay of pyridine leading to its fragmentation (Figure 2(b), red open circles) or to intermolecular proton transfer,^43^ which could be initiated from an Auger final state of pyridine in the presence of a neighboring D_2_O molecule and appears in the time-coincidence map shown in Figure 1 adjacent to the dimer CE line.

## IV Discussion

The present results support a bidirectional ICD mechanism, with a dominant pathway initiated by O 2s ionization of D_2_O and an additional contribution consistent with ICD initiated by N 2s ionization of pyridine. Qualitative insights into the influence of microsolvation in the process can be observed by comparing the pyridine dimer and the pyridine-D_2_O complex. In the pyridine dimer, ICD occurs above 25.5 eV,^12^ whereas in the pyridine-D_2_O complex, it is observed only above 26.5 eV. In both systems, these onset energies are 2–4 eV higher than the theoretically predicted ICD thresholds.^37^ This observation suggests that ICD originating from C 2s vacancies in pyridine, although energetically allowed, is suppressed by competing relaxation pathways such as ultrafast nonadiabatic processes.^44^ Theoretical studies of nonadiabatic dynamics in the outer-valence region of pyridine report lifetimes of 40–100 fs.^45^ However, corresponding investigations for inner-valence ionization are not available. The difference in the onset energies between the pyridine dimer and the pyridine-D_2_O complex indicates that microsolvation influences the relaxation pathways available following inner-valence ionization and consequently affects the onset of the ICD process. Since pyridine serves as a model system for nitrogen-containing aromatic units found in nucleobases, these results show that water opens efficient ICD channels following ionization of its O 2s orbital and support its additional role as an energy acceptor in ICD following inner-valence ionization of the neighboring heteroaromatic molecule.

Before concluding, we would like to note that besides ICD, CE can also be a result of a sequential double ionization (SI), in which the projectile successively removes one electron from the outer-valence orbitals of each monomer. The threshold for this process coincides with that of ICD: twice the lowest ionization potential plus the Coulomb repulsion between the resulting ions. This implies that part of the measured CE events may originate from SI. To estimate its contribution, we compared partial ionization cross sections associated with ICD and SI. The analysis indicates that SI events represent only about 16.6% of the total CE signal (see SM, section VI), confirming that SI plays a minor role in producing pyridine and water ion pairs compared to ICD. Alternative electron-impact double-ionization mechanisms such as intermolecular knockout or shake-off cannot be completely excluded; however, their contributions are expected to be negligible owing to the large intermolecular separation (3–5Å) and the localized character of the molecular orbitals in the pyridine-D_2_O complex.

## V Conclusions

In this work, we investigated the ICD process induced by electron-impact ionization of the pyridine-D_2_O complex using multiparticle momentum coincidence spectroscopy in combination with *ab initio* electronic structure calculations. The onset of the projectile energy-loss spectrum of the CE channel reveals that ICD occurs following population of cationic inner-valence states above 26 eV. Based on the calculated single- and double-ionization potentials, O 2s vacancies in D_2_O and C 2s and N 2s vacancies in pyridine can potentially serve as ICD initiators. The close resemblance between the projectile energy-loss spectra of the ICD and O 2s monomer ionization channels, together with the absence of local Auger decay of D_2_O at the experimental ICD onset, identifies D_2_O as the dominant ICD initiator. The measured ICD electron spectrum, supported by the *ab initio* calculations, provides evidence for an additional pathway initiated by N 2s ionization of pyridine. The reduced contribution of pyridine-initiated ICD can be attributed to competition with Auger decay, which can lead either to fragmentation of the heterocycle or to proton transfer to D_2_O. The experimentally observed ICD onset suggests that C 2s vacancies undergo a different relaxation pathway, possibly involving nonadiabatic dynamics. Similar behavior has been observed and reported for the pyridine dimer, where the ICD onset is likewise associated with the decay of N 2s vacancies rather than C 2s vacancies. These results demonstrate the important role of microsolvation in modifying ultrafast electronic relaxation pathways. Since pyridine serves as a prototypical model for nucleobases, our findings suggest that hydration can profoundly influence radiation-induced decay dynamics in biologically relevant systems.

## Supplementary Material



## Data Availability

The data that support the findings of this study are available from the corresponding author upon reasonable request.

## Notes

Additional details of the experimental and theoretical methods; comparison of spectra for different conformational geometries of Py-D_2_O; and estimation of sequential ionization events (PDF)

## ⊥

Institute of Physics, University of Freiburg, 79104 Freiburg, Germany

## References

[1] Boudaïffa B., Cloutier P., Hunting D., Huels M. A., Sanche L. (2000). Resonant Formation
of DNA Strand Breaks by Low-Energy (3 to 20 eV) Electrons. Science.

[2] Bloß D., Kryzhevoi N. V., Maurmann J., Schmidt P., Knie A., Viehmann J. H., Küstner-Wetekam C., Deinert S., Hartmann G., Trinter F., Cederbaum L. S., Ehresmann A., Kuleff A. I., Hans A. (2025). Interplay of protection
and damage through intermolecular processes in the decay of electronic
core holes in microsolvated organic molecules. Phys. Chem. Chem. Phys..

[3] Laage D., Elsaesser T., Hynes J. T. (2017). Water Dynamics in the Hydration Shells
of Biomolecules. Chem. Rev..

[4] Cederbaum L. S., Zobeley J., Tarantelli F. (1997). Giant intermolecular
decay and fragmentation
of clusters. Phys. Rev. Lett..

[5] Jahnke T., Hergenhahn U., Winter B., Dörner R., Frühling U., Demekhin P. V., Gokhberg K., Cederbaum L. S., Ehresmann A., Knie A., Dreuw A. (2020). Interatomic and Intermolecular
Coulombic Decay. Chem. Rev..

[6] Skitnevskaya A. D., Gokhberg K., Trofimov A. B., Grigoricheva E. K., Kuleff A. I., Cederbaum L. S. (2023). Two-Sided
Impact of Water on the
Relaxation of Inner-Valence Vacancies of Biologically Relevant Molecules. J. Phys. Chem. Lett..

[7] Zhang P., Perry C., Luu T. T., Matselyukh D., Wörner H. J. (2022). Intermolecular Coulombic Decay in Liquid Water. Phys. Rev. Lett..

[8] Richter C., Hollas D., Saak C.-M., Förstel M., Miteva T., Mucke M., Björneholm O., Sisourat N., Slavíček P., Hergenhahn U. (2018). Competition
between proton transfer and intermolecular Coulombic decay in water. Nat. Commun..

[9] Ren X., Wang E., Skitnevskaya A. D., Trofimov A. B., Gokhberg K., Dorn A. (2018). Experimental evidence
for ultrafast intermolecular relaxation processes
in hydrated biomolecules. Nat. Phys..

[10] Hans A., Schmidt P., Kus̈tner-Wetekam C., Trinter F., Deinert S., Bloß D., Viehmann J. H., Schaf R., Gerstel M., Saak C. M., Buck J., Klumpp S., Hartmann G., Cederbaum L. S., Kryzhevoi N. V., Knie A. (2021). Suppression of X-ray-Induced Radiation
Damage to Biomolecules in
Aqueous Environments by Immediate Intermolecular Decay of Inner-Shell
Vacancies. J. Phys. Chem. Lett..

[11] Gao Y., Skitnevskaya A. D., Wang E., Yuan H., Ren X., Lin H., Yan Z., Zhang S., Gu S., Yang B., Fang F., Yan S., Guo D., Zhu X., Zhao D., Shao C., Huang Z., Xue X., Hao X., Zhou J., Zhang T., Li J., Yan X., Wang M., Mao L., Yin D., Tang M., Yuan Y., Yang J., Trofimov A. B., Cederbaum L. S., Kuleff A. I., Ma X., Xu S. (2025). Damaging Intermolecular
Relaxation Processes Initiated by Heavy-Ion Irradiation of Hydrated
Biomolecules. Phys. Rev. X.

[12] Zhou J., Jia S., Xue X., Skitnevskaya A. D., Wang E., Wang X., Hao X., Zeng Q., Kuleff A. I., Dorn A., Ren X. (2024). Revealing
the Role of N Heteroatoms in Noncovalent Aromatic Interactions by
Ultrafast Intermolecular Coulombic Decay. J.
Phys. Chem. Lett..

[13] Ullrich J., Moshammer R., Dorn A., Dörner R., Schmidt L. P. H., Schmidt-Böcking H. (2003). Recoil-ion and electron
momentum spectroscopy: reaction-microscopes. Rep. Prog. Phys..

[14] Ren X., Maalouf E. J. A., Dorn A., Denifl S. (2016). Direct evidence of
two interatomic relaxation mechanisms in argon dimers ionized by electron
impact. Nat. Commun..

[15] Liu C.-T., Lindsay W. T. J. (1970). Vapor pressure of deuterated water from 106 to 300.deg. J. Chem. Eng. Data.

[16] McCullough J. P., Douslin D. R., Messerly J. F., Hossenlopp I. A., Kincheloe T. C., Waddington G. (1957). Pyridine: Experimental and Calculated
Chemical Thermodynamic Properties between 0 and 1500 K.; a Revised
Vibrational Assignment. J. Am. Chem. Soc..

[17] Frisch, M. J. ; Trucks, G. W. ; Schlegel, H. B. ; Scuseria, G. E. ; Robb, M. A. ; Cheese man, J. R. ; Scalmani, G. ; Barone, V. ; Petersson, G. A. ; Nakatsuji, H. ; Li, X. ; Caricato, M. ; Marenich, A. V. ; Bloino, J. ; Janesko, B. G. ; Gomperts, R. ; Mennucci, B. ; Hratchian, H. P. ; Ortiz, J. V. ; Izmaylov, A. F. ; Sonnenberg, J. L. ; Williams-Young, D. ; Ding, F. ; Lipparini, F. ; Egidi, F. ; Goings, J. ; Peng, B. ; Petrone, A. ; Henderson, T. ; Ranasinghe, D. ; Zakrzewski, V. G. ; Gao, J. ; Rega, N. ; Zheng, G. ; Liang, W. ; Hada, M. ; Ehara, M. ; Toyota, K. ; Fukuda, R. ; Hasegawa, J. ; Ishida, M. ; Nakajima, T. ; Honda, Y. ; Kitao, O. ; Nakai, H. ; Vreven, T. ; Throssell, K. ; Montgomery, J. A. ; Peralta, J. E. ; Ogliaro, F. ; Bearpark, M. J. Gaussian Inc 16 Revision C.01; Gaussian Inc: Wallingford CT, 2016.

[18] Pang X., Jiang C., Xie W., Domcke W. (2019). Photoinduced electron-driven
proton transfer from water to an N-heterocyclic chromophore: nonadiabatic
dynamics studies for pyridine-water clusters. Phys. Chem. Chem. Phys..

[19] Mackenzie R. B., Dewberry C. T., Cornelius R. D., Smith C. J., Leopold K. R. (2017). Multidimensional
Large Amplitude Dynamics in the Pyridine-Water Complex. J. Phys. Chem. A.

[20] von
Niessen W., Schirmer J., Cederbaum L. S. (1984). Computational
methods for the one-particle green’s function. Comput. Phys. Rep..

[21] Schirmer J., Barth A. (1984). Higher-order approximations for the
particle-particle propagator. Z. Phys. A Atoms
Nuclei.

[22] Tarantelli F. (2006). The calculation
of molecular double ionization spectra by Green’s functions. Chem. Phys..

[23] Meyer H.-D., Pal S. (1989). A band-Lanczos method
for computing matrix elements of a resolvent. J. Chem. Phys..

[24] Weikert H.-G., Meyer H., Cederbaum L. S., Tarantelli F. (1996). Block Lanczos
and many-body theory: Application to the one-particle Green’s
function. J. Chem. Phys..

[25] Cederbaum L. S., Domcke W., Schirmer J., Niessen W. (1986). Correlation effects
in the ionization of molecules: breakdown of the molecular orbital
picture. Adv. Chem. Phys..

[26] Tarantelli F., Sgamellotti A., Cederbaum L. S. (1991). Many dicationic states and two-hole
population analysis as a bridge to Auger spectra: Strong localization
phenomena in BF_3_. J. Chem. Phys..

[27] Molloy R. D., Danielsson A., Karlsson L., Eland J. H. (2007). Double photoionisation
spectra of small molecules and a new empirical rule for double ionisation
energies. Chem. Phys..

[28] Severs J., Harris F., Andrews S., Parry D. (1993). Triplet-state energies
of H_2_O^2+^: a combined experimental and theoretical
study. Chem. Phys..

[29] Trofimov A. B., Holland D. M. P., Powis I., Menzies R. C., Potts A. W., Karlsson L., Gromov E. V., Badsyuk I. L., Schirmer J. (2017). Ionization
of pyridine: Interplay of orbital relaxation and electron correlation. J. Chem. Phys..

[30] Truong S., Yencha A., Juarez A., Cavanagh S., Bolognesi P., King G. (2009). Threshold photoelectron
spectroscopy of H_2_O and D_2_O over the photon
energy range 12–40 eV. Chem. Phys..

[31] Dempwolff A. L., Paul A. C., Belogolova A. M., Trofimov A. B., Dreuw A. (2020). Intermediate
state representation approach to physical properties of molecular
electron-detached states. II. Benchmarking. J. Chem. Phys..

[32] Trofimov A. B., Zaitseva I. L., Moskovskaya T. E., Vitkovskaya N. M. (2008). Theoretical
investigation of photoelectron spectra of furan, pyrrole, thiophene,
and selenole. Chem. Heterocycl. Compd..

[33] Müller I. B., Cederbaum L. S. (2006). Ionization
and double ionization of small water clusters. J. Chem. Phys..

[34] Stoychev S. D., Kuleff A. I., Cederbaum L. S. (2011). Intermolecular
Coulombic Decay in
Small Biochemically Relevant Hydrogen-Bonded Systems. J. Am. Chem. Soc..

[35] Sisourat N., Kazandjian S., Miteva T. (2017). Probing Conformers of Benzene Dimer
with Intermolecular Coulombic Decay Spectroscopy. J. Phys. Chem. A.

[36] Ren X., Wang E., Zhou J., Jia S., Wang X., Xue X., Dorn A. (2023). Ultrafast molecular dissociation induced by intermolecular
Coulombic decay in water clusters. Phys. Rev.
A.

[37] Mootheril D. M., Skitnevskaya A. D., Ren X., Dogan M., Trofimov A. B., Kuleff A. I., Cederbaum L. S., Pfeifer T., Dorn A. (2025). Enhanced intermolecular
Coulombic decay due to sulfur heteroatoms in thiophene dimer. Commun. Chem..

[38] Vendrell O., Stoychev S. D., Cederbaum L. S. (2010). Generation
of Highly Damaging H2O+
Radicals by Inner Valence Shell Ionization of Water. ChemPhysChem.

[39] Roos A. H., Eland J. H. D., Andersson J., Squibb R. J., Feifel R. (2018). Dissociations
of water ions after valence and inner-valence ionization. J. Chem. Phys..

[40] Mondal S., Wouterlood B., Garcia G. A., Nahon L., Stienkemeier F., Hartweg S. (2026). Dissociative single and double ionization of pyridine. Phys. Chem. Chem. Phys..

[41] Ehrhardt H., Jung K., Knoth G., Schlemmer P. (1986). Differential
cross sections of direct single electron impact ionization. Z. Phys. D:At., Mol. Clusters.

[42] Zobeley J., Santra R., Cederbaum L. S. (2001). Electronic decay in weakly bound
heteroclusters: Energy transfer versus electron transfer. J. Chem. Phys..

[43] Zhou J., Wu L., Belina M., Skitnevskaya A. D., Jia S., Xue X., Hao X., Zeng Q., Ma Q., Zhao Y., Li X., He L., Luo S., Zhang D., Wang C., Trofimov A. B., Slavíček P., Ding D., Ren X. (2025). State- and
time-resolved observation of ultrafast intermolecular proton transfer
in hydrated biomolecules. Nat. Commun..

[44] Galbraith M. C. E., Scheit S., Golubev N. V., Reitsma G., Zhavoronkov N., Despré V., Lépine F., Kuleff A. I., Vrakking M. J. J., Kornilov O., Köppel H., Mikosch J. (2017). Few-femtosecond passage
of conical intersections in the benzene cation. Nat. Commun..

[45] Trofimov A. B., Skitnevskaya A. D., Grigoricheva E. K., Gromov E. V., Köppel H. (2020). Vibronic coupling
in the ground and excited states of the pyridine radical cation. J. Chem. Phys..

