# Supplementary material for: Microsolvation Controls Ultrafast Relaxation Pathways in Hydrated Pyridine
Source: J Phys Chem A. 2026 Oct 7;130(42):8385–93. doi: 10.1021/acs.jpca.6c05980 (PMC13700094; doi:10.1021/acs.jpca.6c05980)
Supplement: Supplementary file 1 [file jp6c05980_si_001.pdf]

Supplementary information: Microsolvation Controls Ultrafast Relaxation Pathways in Hydrated Pyridine

Deepthy Maria Mootheril[1], Anna D. Skitnevskaya[2], Xueguang Ren[3], Alexander I. Kuleff[4], Alexander B. Trofimov[2], Lorenz S. Cederbaum[4], Thomas Pfeifer[1], and Alexander Dorn[1]

[1] *Max-Planck-Institut für Kernphysik, Saupfercheckweg 1, 69117 Heidelberg, Germany*
[2] *Federal Research Center, A.E. Favorsky Irkutsk Institute of Chemistry, Siberian Branch of the Russian Academy of Sciences, 664033 Irkutsk, Russia*
[3] *MOE Key Laboratory for Nonequilibrium Synthesis and Modulation of Condensed Matter, School of Physics, Xi'an Jiaotong University, 710049 Xi'an, China*
[4] *Theoretische Chemie, Physikalisch-Chemisches Institut, Universität Heidelberg, 69120 Heidelberg, Germany*

I Procedure for the correction of time-of-flight

In the experiment, ions are extracted from the interaction region using a high electric field (∼23 V/cm) applied in a pulsed mode, which is ramped up approximately 500 ns after the electrons are detected. Because the electric field rises to the maximum value over a finite time (∼ 0.6 μs), not all fragments experience the same average field strength. Fragments emitted in the $+z$ and $-z$ directions therefore encounter different effective extraction fields. Those emitted in the $-z$ direction (toward the ion detector) experience a smaller average field than the maximum value, whereas fragments emitted in the $+z$ direction encounter a field closer to the final maximum electric field. As a result, ions arrive at the detector later than expected, producing a tilt in the diagonal coincidence line of the ion–ion time-of-flight distribution. This effect is particularly pronounced for lighter fragments such as D_2O^+, which may leave the acceleration region before the electric field reaches its maximum value.

To correct for this effect, a time-of-flight correction is applied to the Coulomb-explosion fragments according to

$$t_{corr} = t - C\frac{|t - t_{p_z=0}|}{|t_{p_z=0} - t_{p_z(min/max)}|} \tag{1}$$

where t is the measured time-of-flight, C is the correction factor, $t_{p_z(min/max)}$ is the time-of-flight for fragments emitted along the spectrometer axis (±z direction) and $t_{p_z=0}$ is the time-of-flight of the fragments emitted in the direction perpendicular to the spectrometer axis.

II Procedure for determination of energy shift to ADC calculations

II.1 ADC(3) for single ionization

An empirical energy correction was applied to align the ADC-calculated ionization spectra of the Py-D_2O dimer with the experimental results. This adjustment compensates for the finite basis set limitations and the systematic errors inherent to the computational methodology used. In ADC(3) single-ionization spectra, the correction remains small (∼0.2–0.3 eV) for one-hole (1h) states dominating the outer-valence region. In contrast, for the higher-lying cation-radical satellite states of two-hole-one-particle (2h-1p) character that prevail in the inner-valence region, the deviation is larger because such states are described only at first order within the ADC(3) scheme.

The required energy corrections for pyridine and D_2O were obtained by aligning the theoretical spectra with the experimental photoelectron data for the corresponding monomers, as shown in Figs. S1 and S2. For pyridine, two prominent bands in the inner-valence region, corresponding to the C 2s and N 2s vacancies, were compared with the experimental results [1], yielding average energy shifts of 1.2 eV and 1.5 eV, respectively. For D_2O, comparison between the calculated single-ionization spectrum and the experimental photoelectron spectrum [2] (see Fig. S2) indicated a red shift of approximately 1.1 eV, which was applied to align the calculated spectrum with experiment.

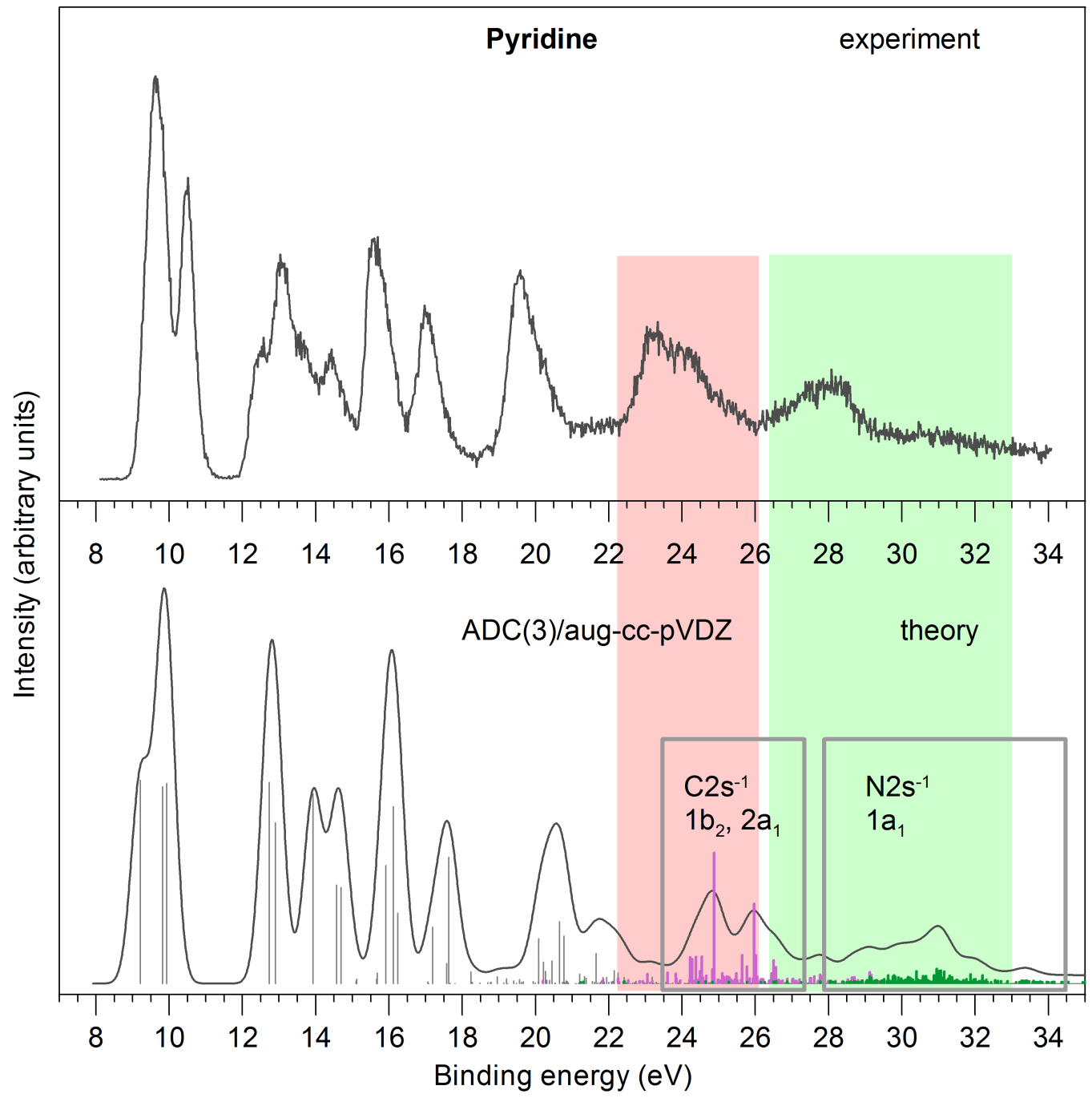

Figure S1: Upper panel: Experimental photoionization spectrum of pyridine taken from ref. [1]. Lower panel: Theoretical single-ionization spectrum of the pyridine monomer, calculated with ADC(3)/aug-cc-pVDZ. The grey boxes indicate the bands corresponding to C 2s and N 2s vacancies. Individual C $2s^{-1}$ and N $2s^{-1}$ states are shown as pink and green sticks, respectively, while the corresponding regions in the experimental spectrum are indicated by the red and green shaded areas.

II.2 ADC(2) for double ionization

For the ADC(2) calculations of double-ionization potentials (DIPs), the required energy correction is typically larger and can reach up to about 2 eV. Table S1 provides an example illustrating how these shifts were derived by comparing the experimental and theoretical double-ionization thresholds of pyridine and water. Based on this comparison, a blue shift of 0.7 eV was determined for pyridine and 1.3 eV for water. Consequently, an average blue shift of 1 eV was applied to the calculated double-ionization spectra of the Py-D_2O dimer.

Table S1: Calculated energies of the dicationic states using ADC(2) theory and their comparison with experimental values used to determine the energy shift.

DIP	Pyridine (eV)	Water (eV)
Experiment	25.5 [3]	40.0 [4]
ADC(2)/aug-cc-pVDZ	24.8	38.7
ΔE	0.7	1.3

III Extraction of experimental ICD electron energy spectrum

Extraction of the ICD electron spectrum (Fig. 3b) relies on subtraction of the O^+ reference channel, assuming it represents both water and pyridine ionization and that normalization above 25 eV removes non-ICD contributions. For given projectile energy E_0 and ionization potential/binding energy IP, the energy sharing of the final state electrons is E_0 - IP = E_{sc} + E_{ej}. Therefore, the final state electron energy distribution (single differential cross section) is mirror symmetric to (E_0 − IP)/2. Thus, for similar energy loss spectra of different species at higher energy they should have also similar ejected electron spectra at low energy. Considering ionization of two different molecules and ionization of orbitals with similar binding energies in the two different molecules. Then the ejected electron energy

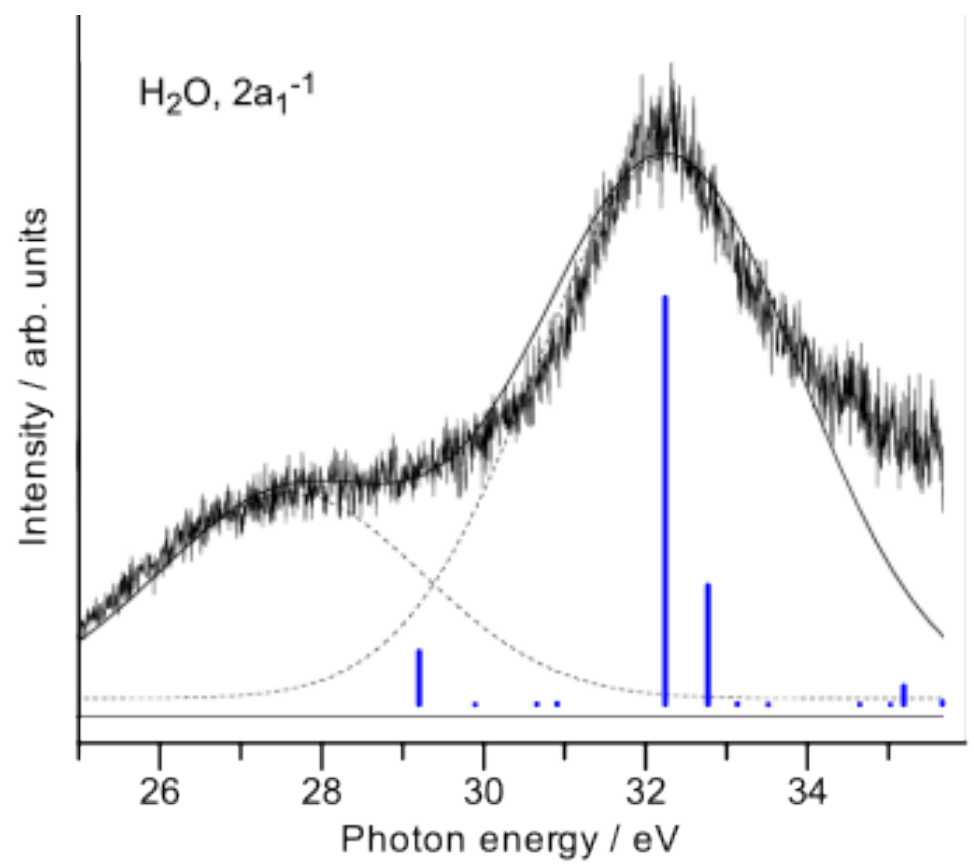

Figure S2: Experimental [2] and theoretical ionization spectra (blue sticks) of the water monomer. The theoretical spectrum has been calculated using ADC(3)/aug-cc-pVDZ and shifted by 1.1 eV to lower energies to match the experimental data.

distributions are very close to each other (for the same projectile impact energy). The reasoning is as follows: The ionized electron momentum distribution originates/results from the sum of (a) the momentum transferred by the scattered projectile and (b) the initial momentum in the bound orbital. Since the projectile beam properties are identical for both molecules we assume that in ionization the momentum transfer distribution to a bound electron is very similar for both molecules. The momentum distribution in the bound state (b) is closely related to the orbital binding energy. According to the Virial Theorem the average kinetic energy in a Coulomb potential is equal to half the magnitude of its potential energy. Therefore, the bound orbital momentum distributions for different orbitals with similar binding energies are also similar. As result in good approximation the emitted electron energy spectra are similar for the cases discussed in the manuscript.

IV Comparison of ICD electron spectra with different contributions from O 2s and N 2s

Fig. S3 presents the experimental and theoretical ICD-electron spectra of the pyridine-water system. From the upper panel of the figure, one can see that the experimental spectrum shows a pronounced peak at ∼1 eV, a plateau between 2 and 4 eV, and a smaller peak at ∼6 eV. While the O 2s spectrum reproduces the peaks at 1 and 6 eV, it shows an additional peak at 4 eV instead of the experimental plateau; conversely, the N 2s spectrum reproduces the plateau between 2 and 4 eV but lacks the peak at 6 eV. This indicates that the experimental spectrum arises from a combination of both decay channels. Different proportions of the two channels are shown in the lower panel of Fig. S3.

V Comparison of spectra with different conformational geometries of Py-D_2O

Figure S4 compares simulated single- and double-ionization spectra, as well as the ICD electron spectra for different conformers of the Py-D_2O dimer [5]. The conformers differ mainly in the relative orientation of the water molecule with respect to the pyridine ring. As shown in the IP spectra (bottom panels of Figs. S4a and S4b), the overall shapes and intensities are nearly identical across conformers, indicating that the decay probabilities and energies are largely unaffected by the orientation of the water molecule. The same trend is observed in the DIP spectra (middle panels), which display only minor variations between geometries. Consequently, the ICD electron spectra (top panels) also exhibit very similar profiles, suggesting that the electronic decay dynamics in the dimer are essentially independent of its specific hydrogen-bonding arrangement.

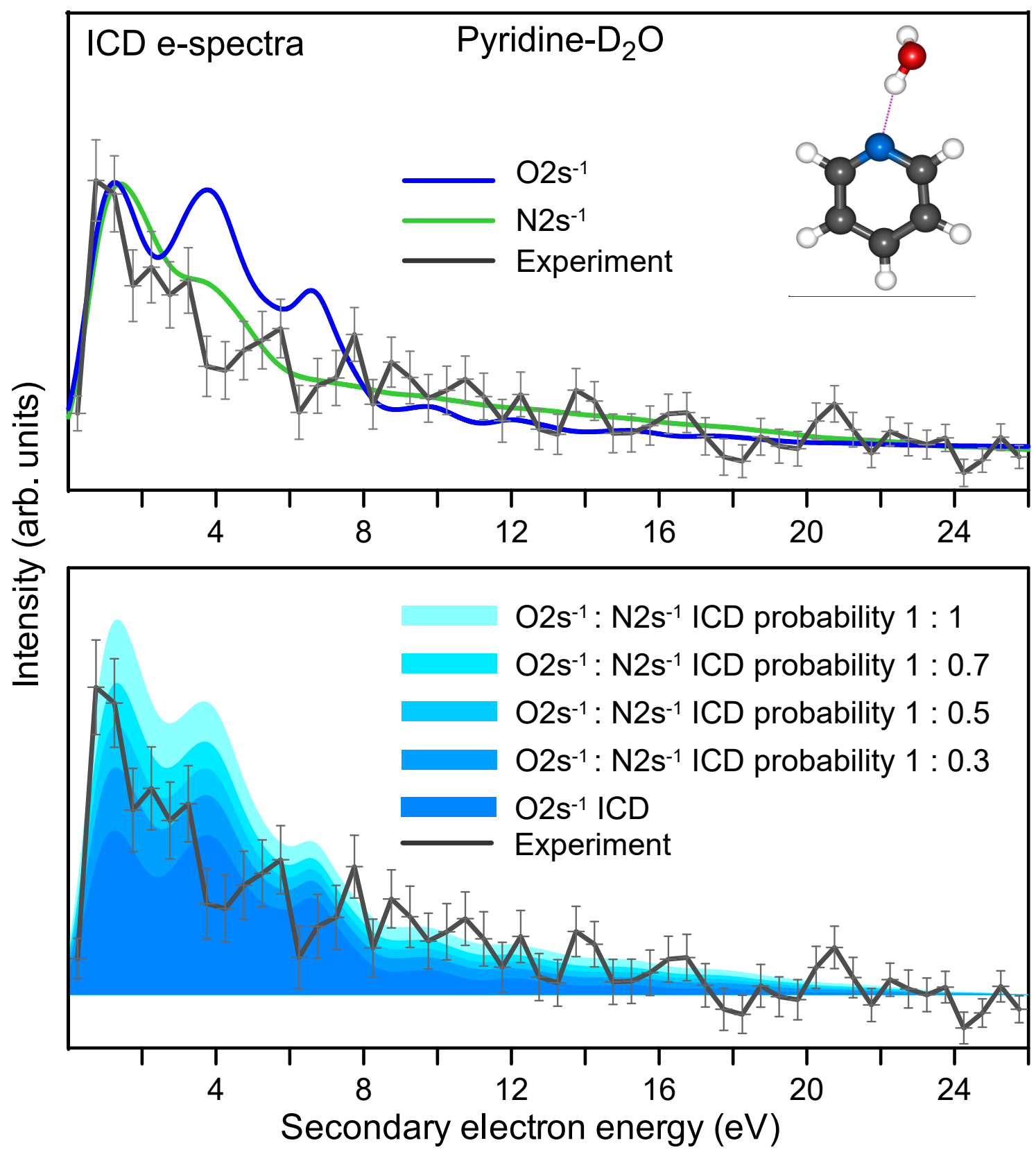

Figure S3: ICD electron spectra of the pyridine-water system. Upper panel: simulated electron energy spectra produced via ICD from the O 2s vacancy (blue) and the N 2s vacancy (green) both normalized to the intensity of the experimental spectrum (black). Lower panel: experimental spectrum (black) and ICD electron spectra computed considering the probability of ICD decay for O 2s^{-1} states to be 100%, while that for N 2s^{-1} states was varied from 0 to 100%; no additional scaling was applied. All the spectra were computed using the procedure described in the methods section from the IP states having energies higher than 26.5 eV. For better comparison with the experimental data only electrons having energies above 0.5 eV were included and the convolution was done with Gaussian with FWHM=1 eV.

VI Estimation of sequential ionization events

The sequential ionization (SI) contribution was estimated by treating it as a two-step process: removal of one electron from the first monomer, followed by ionization of the second. The probability of the latter step was approximated from the ratio of the relevant ionization cross section to the surface area of a sphere with radius equal to the equilibrium intermolecular distance ($R = 4.12$ Å):

$$\sigma_{\mathrm{SI}} = \sigma_{Py^+}(E_0 = 103)\frac{\sigma_{\mathrm{D_2O^+}}(E_1 = 93.4)}{4\pi R^2} + \sigma_{\mathrm{D_2O^+}}(E_0 = 103)\frac{\sigma_{Py^+}(E_1 = 90.4)}{4\pi R^2} \qquad (2)$$

The partial cross sections used in this expression are $\sigma_{Py^+}(103\ \mathrm{eV}) = 4.47\times10^{-16}\ \mathrm{cm}^2$, $\sigma_{\mathrm{D_2O^+}}(93.4\ \mathrm{eV}) = 1.38\times10^{-16}\ \mathrm{cm}^2$, $\sigma_{\mathrm{D_2O^+}}(103\ \mathrm{eV}) = 1.374\times10^{-16}\ \mathrm{cm}^2$, and $\sigma_{Py^+}(90.4\ \mathrm{eV}) = 4.6\times10^{-16}\ \mathrm{cm}^2$ [6, 7], giving $\sigma_{\mathrm{SI}} = 5.855\times10^{-18}\ \mathrm{cm}^2$.

For comparison, the ICD channel cross section was obtained by assuming that CE originates from ionization of the O 2s (2a$_1$) orbital in D_2O. Using the O^+ production cross section $\sigma_{\mathrm{O^+}}(103\ \mathrm{eV}) = 7.452\times10^{-18}\ \mathrm{cm}^2$ and applying a branching ratio of 24% for the 2a$_1$ orbital [7, 8], the corresponding value is $\sigma_{\mathrm{O}2s^{-1}} = 2.98\times10^{-17}\ \mathrm{cm}^2$. From this comparison, SI is estimated to account for only ∼16.6% of the total CE yield, confirming its minor role relative to ICD. No strong energy dependence of the SI contribution is expected within the investigated energy range, as the relevant electron-impact ionization cross sections vary only weakly with the scattered-projectile energy. Therefore, residual SI contributions are not expected to account for the observed low-energy electron enhancement.

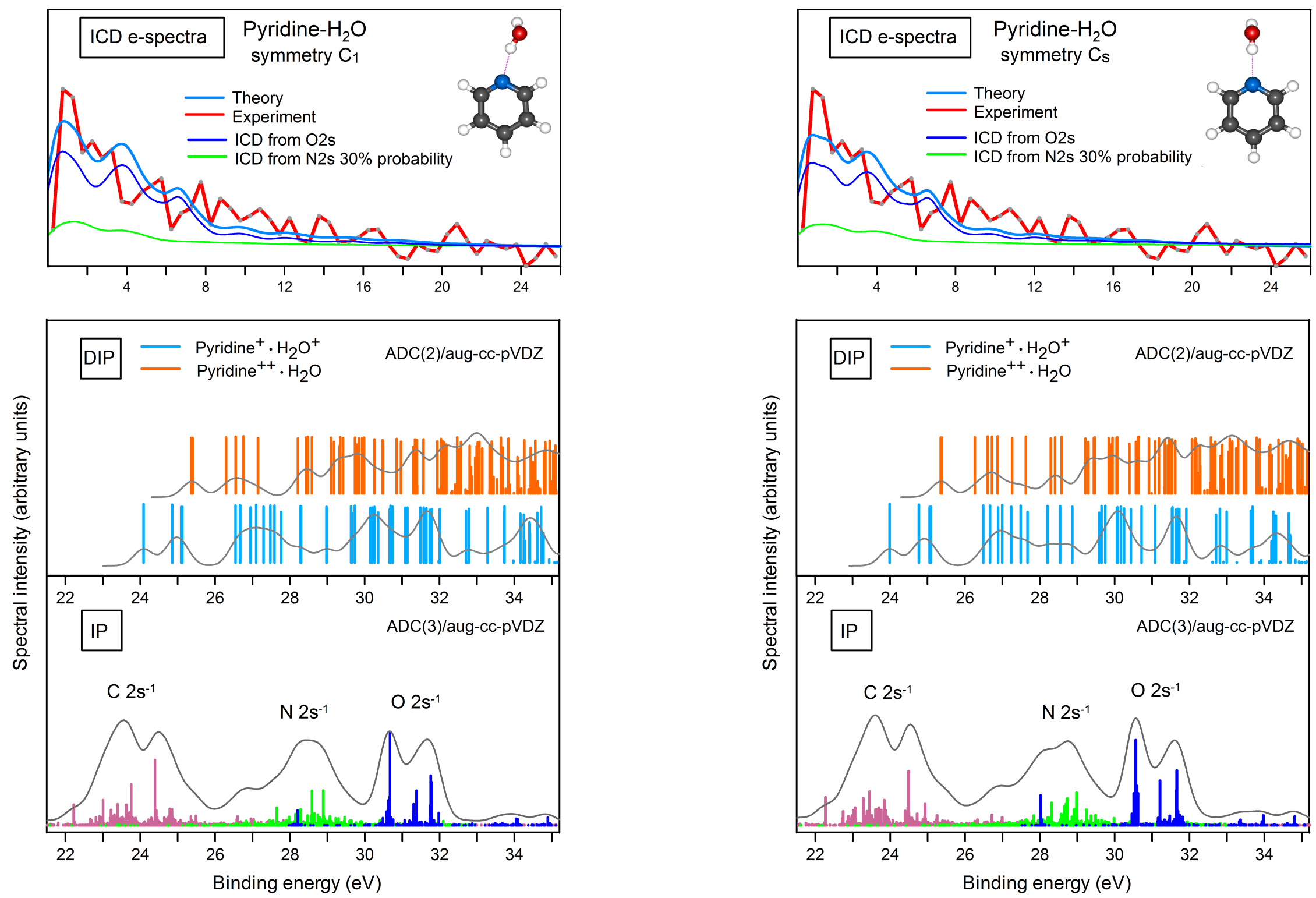

(a) (b)

Figure S4: **Comparison between different conformers of the Py-D_2O dimer.** Single-ionization (IP, bottom panel), double-ionization (DIP, middle panel), and ICD spectra (top panel) are shown for conformers with (a) C_1 and (b) C_s symmetry.

References

[1] Trofimov, A. B.; Holland, D. M. P.; Powis, I.; Menzies, R. C.; Potts, A. W.; Karlsson, L.; Gromov, E. V.; Badsyuk, I. L.; Schirmer, J. Ionization of pyridine: Interplay of orbital relaxation and electron correlation. *The Journal of Chemical Physics* **2017**, *146*, 244307.

[2] Truong, S.; Yencha, A.; Juarez, A.; Cavanagh, S.; Bolognesi, P.; King, G. Threshold photoelectron spectroscopy of H2O and D2O over the photon energy range 12–40eV. *Chemical Physics* **2009**, *355*, 183–193.

[3] Molloy, R. D.; Danielsson, A.; Karlsson, L.; Eland, J. H. Double photoionisation spectra of small molecules and a new empirical rule for double ionisation energies. *Chemical Physics* **2007**, *335*, 49–54.

[4] Severs, J.; Harris, F.; Andrews, S.; Parry, D. Triplet-state energies of H_2O^{2+}: a combined experimental and theoretical study. *Chemical Physics* **1993**, *175*, 467–473.

[5] Mackenzie, R. B.; Dewberry, C. T.; Cornelius, R. D.; Smith, C. J.; Leopold, K. R. Multidimensional Large Amplitude Dynamics in the Pyridine–Water Complex. *The Journal of Physical Chemistry A* **2017**, *121*, 855–860.

[6] Jiao, C.; DeJoseph, C.; Lee, R.; Garscadden, A. Kinetics of electron impact ionization and ion-molecule reactions of pyridine. *International Journal of Mass Spectrometry* **2006**, *257*, 34–40.

[7] Straub, H. C.; Lindsay, B. G.; Smith, K. A.; Stebbings, R. F. Absolute partial cross sections for electron-impact ionization of H_2O and D_2O from threshold to 1000 eV. *The Journal of Chemical Physics* **1998**, *108*, 109–116.

[8] Roos, A. H.; Eland, J. H. D.; Andersson, J.; Squibb, R. J.; Feifel, R. Dissociations of water ions after valence and inner-valence ionization. *The Journal of Chemical Physics* **2018**, *149*, 204307.
